# Influence of Galactooligosaccharides on the Positive Effect of Plant Sterol-Enriched Beverages on Cardiovascular Risk and Sterol Colon Metabolism

**DOI:** 10.1021/acs.jafc.1c06120

**Published:** 2022-01-11

**Authors:** Virginia Blanco-Morales, Ramona de los Ángeles Silvestre, Elena Hernández-Álvarez, Encarnación Donoso-Navarro, Amparo Alegría, Guadalupe Garcia-Llatas

**Affiliations:** † Nutrition and Food Science Area, Faculty of Pharmacy, University of Valencia, Avda. Vicent Andrés Estellés s/n, Burjassot, Valencia 46100, Spain; ‡ Clinical Biochemistry, Hospital Universitario Puerta de Hierro-Majadahonda, Universidad Autónoma de Madrid, C/Manuel de Falla, 1, Madrid 28222, Spain

**Keywords:** cholesterol absorption, clinical trial, fecal sterols, hypercholesterolemia, lathosterol, non-cholesterol sterols, phytosterols, post-menopausal women

## Abstract

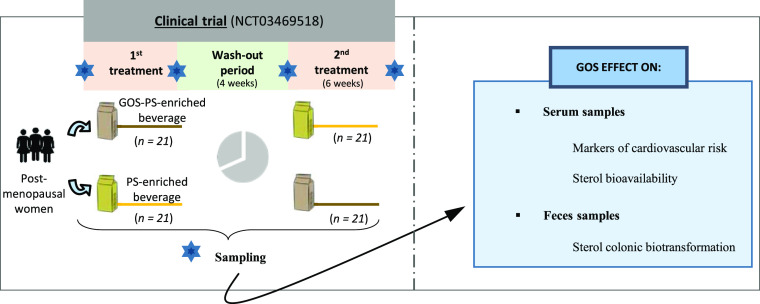

In the present study, the impact of galactooligosaccharide (GOS) addition to a plant sterol (PS)-enriched beverage on the hypocholesterolemic effect and on the bioavailability and colonic metabolization of sterols was evaluated. A crossover trial was undertaken in postmenopausal women who intook a PS-enriched (2 g PS/day) or PS–GOS-enriched beverage (2 g PS/day and 4.3 g GOS/day) for 6 weeks. The presence of GOS did not modify the hypocholesterolemic effect of the PS-enriched beverage (total- and low-density lipoprotein-cholesterol reductions) or sterol bioavailability (increments of serum markers of dietary PS intake and of cholesterol synthesis). The consumption of both beverages led to an increase of sterol and metabolite excretion (with the exception of coprostanol, which decreased) and to slight changes in women’s capacities for sterol conversion, regardless of the GOS presence. This study demonstrates the suitability of simultaneous enrichment with PS and GOS in milk-based fruit beverages, considering their hypocholesterolemic effect.

## Introduction

According to the European Atherosclerosis Society Consensus Panel, individuals with hypercholesterolemia, at intermediate or low cardiovascular risk, and not qualified for drug treatment ought to consider the consumption of plant sterol (PS)-enriched foods.^1^ The hypocholesterolemic effect, obtained with a daily intake of 1.5–3 g of PS, is recognized as a health claim related to the reduction in disease risk.^2−4^

In postmenopausal women, risk factors of cardiovascular disease (total- and low-density lipoprotein (LDL)-cholesterol levels) could be increased, favoring atherosclerotic processes.^5,6^ In this context, in previous works of our group, a positive synergistic effect on cardiovascular risk of β-cryptoxanthin (β-Cx) and PS added to milk-based fruit beverages, without^7^ or with milk fat and milk fat globule membrane (MFGM),^8^ has been demonstrated in postmenopausal women with moderate hypercholesterolemia not pharmacologically treated. Moreover, the bioavailability of sterols has been evaluated by determining the serum concentrations of PS and cholesterol precursors.^8,9^

On the other hand, nonabsorbed PS (β-sitosterol, campesterol, and stigmasterol) can be transformed, in the same way as cholesterol, into their corresponding metabolites (ethyl and methylcoprostanol or ethylcoprostanol and subsequently to ethyl and methylcoprostanone or ethylcoprostanone).^10,11^

As far as we are aware, only two studies have assessed the influence of the intake of high doses of PS, from enriched foods, on the excretion of sterols and their metabolites. One of them conducted with normolipidic subjects whose diet has been enriched or not enriched with PS-enriched margarine (8.6 g/day)^12^ exceeded the content of PS established by the European Commission.^13^ The other one was carried out by our research group in postmenopausal women with moderate hypercholesterolemia who ingested milk-based fruit beverages containing β-Cx enriched or not enriched with PS (2 g/day).^14^

**Figure 1:**
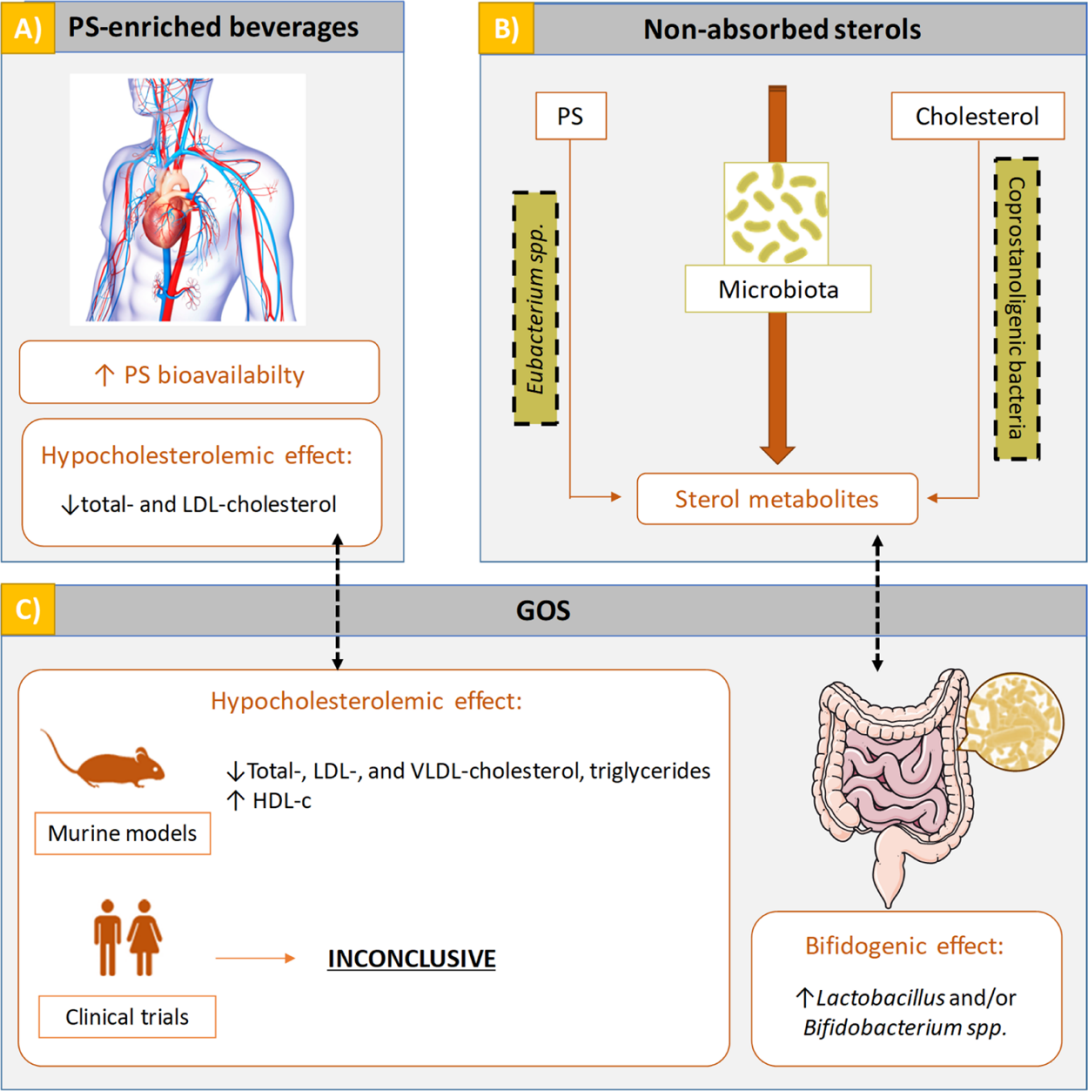
Possible interaction between PSs and GOSs (absorption and metabolism). (A) Hypocholesterolemic effect after the regular consumption of PS-enriched milk-based fruit beverages has been confirmed in postmenopausal women as well as an increase in the bioavailability of PS.^8,9^ (B) Nonabsorbed sterols (PS and cholesterol) are susceptible to biotransformation by the action of the microbiota into sterol metabolites. Among the microbial species associated with this process, *Eubacterium* spp. has been the only one related to PS metabolism. With respect to cholesterol, different bacteria have been associated (*Eubacterium* spp., *Bacteroides* spp., *Bifidobacterium* spp., *Clostridium* spp., and *Lactobacillus* spp.), all of which are referred to as coprostanoligenic bacteria.^11^ (C) In the present study, the addition of GOS to PS-enriched beverages was proposed aiming at improving the functionality of this food matrix. On the one hand, the major health benefit associated with the consumption of GOS is their ability to selectively stimulate the growth of specific members of the gut microbiota. In particular, they are highly specific in increasing the microbial population of *Bifidobacterium* spp. and *Lactobacillus* spp.,^15^ coprostanoligenic bacteria as abovementioned. We hypothesize that this modulation of the microbiota exerted by the presence of GOS could modify sterol metabolism. In the other hand, information regarding the effect of GOS on the serum lipid profile is scarce. Studies in murine models have shown that consumption of GOS for 3–8 weeks is able to improve the lipid profile.^16−18^However, in clinical trials lasting 6–12 weeks, the results are inconclusive.^19,20^ Moreover, their possible interference with PS absorption is unknown. Thus, the present work sheds light on the influence of the prebiotic upon hypocholesterolemic effect of the PS-enriched beverages and sterol bioavailability.

Galactooligosaccharides (GOSs), which can be easily incorporated into milk-based fruit beverages due to their technological properties, could enhance the already demonstrated effect of PS-enriched beverages by contributing beneficial effects at the intestinal level (see Figure 1).^15^ Information regarding the effect of GOSs on the serum lipid profile is scarce and inconclusive involving murine models^16−18^ or humans.^19,20^ It has been confirmed that the addition of GOSs to this type of beverage does not affect the bioaccessibility of total PS after simulated gastrointestinal digestion,^21^ but this remains to be confirmed by *in vivo* studies in order to ensure their functionality. As far as we know, there are no data available about the effect of simultaneous intake of PS and GOS on PS bioavailability or the hypocholesterolemic effect of these beverages in humans.

Therefore, the objective of this work is to evaluate whether the regular consumption of GOSs in a PS-enriched milk-based fruit beverage by postmenopausal women modifies the *in vivo* bioavailability of PS and the hypocholesterolemic effect/markers of cardiovascular risk as well as the colonic metabolization of sterols. A crossover clinical study, in which each subject has their own control, was carried out for this purpose, thus reducing possible individual variability that could mask the effect studied. The measurement of primary (serum levels of sterols) and secondary (serum lipid and sterol fecal profiles) outcomes was used for this aim.

## Materials and Methods

### Samples

Two PS-enriched skimmed milk-based fruit beverages (2 g PS/250 mL) enriched with or without GOS (4.3 g/250 mL) were manufactured specifically for this study. Both beverages have been elaborated under the same conditions and had similar ingredients: skimmed milk with the addition of milk fat and whey protein concentrate enriched with MFGM (49%), mandarin juice from concentrate (45%), banana puree (4%), microencapsulated free microcrystalline PS from tall oil (Lipophytol 146 ME Dispersible, Lipofoods, https://www.lipofoods.com/en/products/lipophytol.html) (as a water-dispersible source of PS), GOS syrup (Vivinal GOS from FrieslandCampina Ingredients), and pectins. The production of this type of beverage has been published in previous works.^21,22^ The functionality of the ingredients that integrate the beverage (β-Cx and PS) is based on previous clinical studies,^7,8,14^ and the GOS dose is based on an *in vitro* bioaccessibility study,^21^ all of which were carried out by our research group. The energy and nutritional information, per 100 mL, for the GOS–PS-enriched and PS-enriched beverages were, respectively, energy (kcal): 78 or 80; fat (g): 2 or 2.2; carbohydrates (g): 11.3 and 15.6; protein (g): 2.7 and 2.7, and fiber (g): <0.5. PS contents in both beverages were analyzed by gas chromatography-flame ionization detection (GC-FID) described elsewhere.^22^ Relative percentages of PS were β-sitosterol: 81.0%, sitostanol: 11.2%, campesterol: 6.1%, campestanol: 1.0%, and stigmasterol: 0.7%.

### Clinical Study/Intervention Study

A single and combined randomized, double-blind, crossover trial was carried out in postmenopausal women with mild hypercholesterolemia (200–239 mg/dL) according to the guidelines of the American Heart Association^23^ (ClinicalTrials.gov number NCT03469518). The clinical study took place in the Vitamins Unit of the Department of Clinical Biochemistry of Hospital Universitario Puerta de Hierro-Majadahonda (Madrid, Spain). The study protocol was approved by the Clinical Research Ethics Committee of the aforementioned hospital, and all participants gave their written consent.

The inclusion criteria were as follows: age (45–65 years), body mass index (BMI) < 35 kg/m^2^, amenorrhoea over 12 months, nondieting and nonintake of vitamin D, calcium, ω-3 fatty acids, PS or vitamin-enriched foods, or supplements or other dietary bioactive components. Exclusion criteria were use of vitamins, hormone replacement therapy, fibrates, statin ezetimibe, polyunsaturated fatty acids, and a weight-loss diet, as well as acute inflammation, chronic medication, and infection or intercurrent illness capable of affecting the bioavailability or status of the compounds of interest.

A total of 42 healthy postmenopausal women were finally included in the study and were sequentially numbered from 1 to 42. The sample size was calculated considering the total PS and cholesterol results obtained in a previous clinical trial (ClinicalTrials.gov number NCT01074723). Taken from previous assumption, we chose the most conservative option to ensure the detection of a 7% decrease in cholesterol levels in mildly hypercholesterolemic subjects (e.g., 15 mg/dL) with a type I error of 0.05 and a statistical power of 80%. Moreover, allowing for a 45% rate of the Western population likely to present polymorphisms implicated in the cholesterol absorption process and assuming a drop-out rate of 10%, the final required sample size was stipulated to comprise 42 persons.

**Figure 2:**
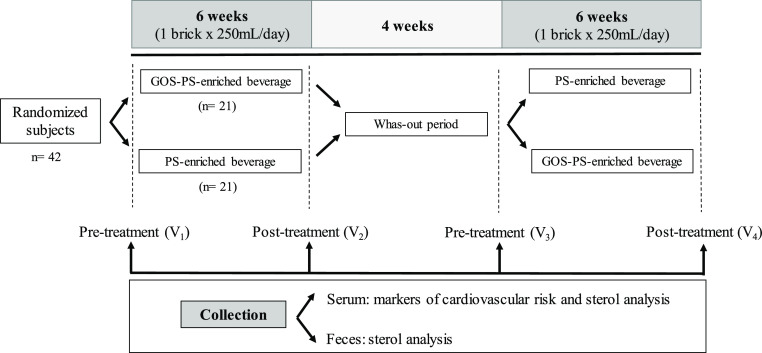
Overview of the study.

The two beverages, of the same color and taste, were filled in 250 mL cartons, being indistinguishable, with different anonymous labeling (A or B). During the 6 week intervention period, 21 subjects consumed the PS-enriched beverage, while 21 subjects consumed the PS-enriched beverage with GOS, both on a daily basis. After a 4 week wash-out period, the type of beverage to be consumed during another 6 week period was changed in-between groups. A diagram of the clinical trial is shown in Figure 2.

The volunteers were allocated for intervention at random order, using a computer-generated pseudo-random number table. A member of the research team (not involved in subject selection) requested each subject to randomly select one of a series of opaque sealed envelopes containing identification of the type of beverage. After opening the selected envelope, the type of beverage (with or without GOS) assigned to each subject was recorded and a pack with enough cartons to cover the first experimental period (6 weeks) was provided. It was also ensured that each subject was assigned to the other study group (with or without GOS) following the corresponding washout period. The details of group assignment were kept in a sealed envelope that was opened at the end of the complete experimental period. Neither the subjects nor the rest of the research team knew about subject assignment during the experimental period. The participants were provided with a list of foods and beverages rich in β-Cx that were to be avoided and were requested not to change their usual diet or physical activity. They were also instructed to record any side effects during the study and to complete a semiquantitative food frequency questionnaire (FFQ)^14^ at the end of each intervention period. Compliance was confirmed at the end of each intervention period by requesting the number of noningested cartons.

### Blood and Feces Sampling

Sample collection was performed before and after each 6 week treatment period (see Figure 2), when a centralized service assigned a 7-digit identification number to each subject (following the usual practice for all hospital patients), and a member of the research team supervised the samples from each subject, which were collected in sterile plastic containers and stored at −20 °C until analysis. Only the nurse or laboratory technicians knew the assigned number of the samples and were unaware of which treatment was received by the participants.

### Markers of Cardiovascular Risk

To confirm mild hypercholesterolemia (basal level) and to evaluate the effect of the PS-enriched beverages (with or without GOS) on the lipid profile, total and high-density lipoprotein (HDL)-cholesterol were determined using an automated routine method (Advia 2400 Clinical Chemistry System, Siemens Healthineers).^7,8^ Periodically, these analyses were subjected to External Quality Assurance Program of the Spanish Society of Medicine of the SEQC-ML Laboratory, which implemented a quality management system in accordance with the UNE-EN ISO 9001 standard certified by AENOR. The Friedewald equation was used to estimate the LDL-cholesterol concentration.^24^

### Serum Biomarkers: Sterol Analysis

PS (campesterol, stigmasterol, and β-sitosterol) and cholesterol precursors (desmosterol and lathosterol) and metabolite (cholestanol) in serum samples were analyzed following a previously validated methodology.^25^ Briefly, serum samples (100 μL), added with epicoprostanol as the internal standard (2 μg), were saponified with 1 mL of potassium hydroxide ethanolic solution (0.75 M) at 65 °C for 1 h. Then, the unsaponifiable fraction was extracted with different washes of *n*-hexane and centrifugation (18 °C/3600 rpm/10 min). The organic phase obtained was derivatized with 200 μL of 10:3 (v/v) *N*,*O*-bis(trimethylsilyl)-trifluoroacetamide [1% trimethylchorosilane]/pyridine at 65 °C for 1 h. The trimethylsilyl ether derivatives obtained were dissolved and filtered (Millex-FH filter unit, 0.45 μm Millipore, Milford, MA) with *n*-hexane, evaporated, and dissolved in 50 μL of *n*-hexane. A total of 1 μL of derivatized samples was injected into the GC–FID system.

### Fecal Sterols and Their Metabolites

This determination was carried out following the methodology validated by Cuevas-Tena et al. (2017).^26^ The procedure applied was the same as for the determination of serum sterols, with the exception that feces samples require a pre-treatment step. In this regard, fresh fecal samples were stored at −20 °C and subsequently freeze-dried (Sentry 2.0, Virtis SP Scientific) and crushed in a glass mortar and stored at −20 °C until analysis. Then, approximately 30 mg of freeze-dried feces were dispersed in 5 mL of Milli-Q water, sonicated (20 min), and allowed to stand for 2 h at room temperature. The analysis was carried out on aliquots of different volumes depending on the concentration of the sterol to be analyzed (25, 100, and 500 μL) and using 20 μg of 5α-cholestane as the internal standard. The saponification, extraction of the unsaponifiable fraction, and derivatization steps were performed as described in the previous section, and 1 μL of derivatized samples was injected into the GC–MS system.

### Statistical Analysis

Comparison of the total cholesterol and HDL- and LDL-cholesterol levels was performed by paired *t*-test for parametric variables. A value of *p* < 0.05 will be considered statistically significant, and Medcalc program (MedCalc Version 11.4.2.0) was used.

To confirm the use of nonparametric test, the normal distribution of sterol and metabolite contents in serum and feces was evaluated using the Shapiro–Wilk test. Wilcoxon test was used in order to detect statistically significant differences in serum sterol (cholesterol precursors and metabolite and PS) and in fecal sterol contents between pre-treatment and post-treatment and changes in values (absolute and percentage) between beverages (PS-enriched or GOS–PS-enriched). Univariate correlations between serum cholesterol levels and fecal cholesterol/coprostanol ratio or fecal cholesterol percentage of conversion after the intake of both beverages were investigated using the Spearman coefficient. In all cases, a level of *p* < 0.05 was used as the criterion for statistical significance, and the Statgraphics Centurion XVI.I statistical package was used. The analysis of all samples was performed in triplicate.

## Results and Discussion

### Progress of the Study

**Figure 3:**
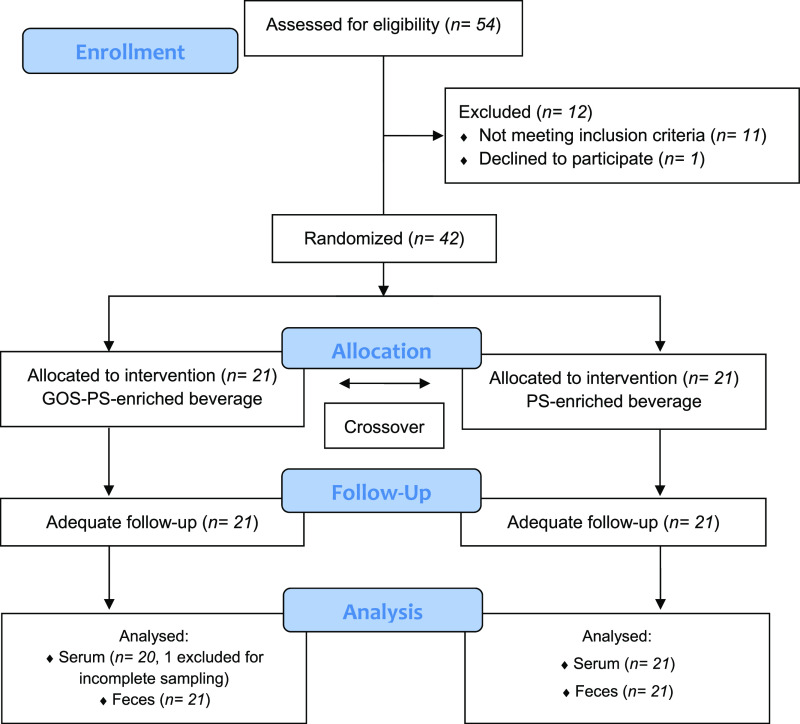
Participant flow.

Participant flow, which started in March 2017 and was completed in June 2017, is shown in Figure 3. 54 postmenopausal women were contacted for participation and interviewed in order to confirm that they met the inclusion criteria (enrollment). 12 were excluded; 42 women participated, and they were randomly assigned. In the analysis phase of sterol determination in serum samples, a subject was excluded due to incomplete sampling in the second intervention period. The women who finally participated in the study had an average age of 58.4 ± 4.1 years (range 45–67) and presented untreated mild hypercholesterolemia (229.8 ± 25.2 g/dL) with a BMI of 24.5 ± 3.2 kg/m^2^. At the end of the study, the participants reported not having detected any differences in the organoleptic properties of the two beverages in the FFQ; neither did they express any body weight changes.

### Impact of GOS on the Lipid Profile

#### Hypocholesterolemic Effect

Contents of total, HDL-, and LDL-cholesterol at pre-treatment and posttreatment are reported in Table 1. As it can be observed, the addition of GOS to these beverages did not modify the hypocholesterolemic effect. The consumption of the beverages exerted similar (*p* > 0.05) decreases in total (4.7–5.1%) and LDL-cholesterol (7.6–9.0%) without significant changes in HDL-cholesterol. Accordingly, a clinical study carried out with healthy adults showed that the intake of GOS (in powder form) at a similar dose of our study (5.5 *vs* 4.3 g/day, respectively) during 10 weeks did not modify cholesterol and HDL plasma cholesterol.^19^ However, in a study concerning adults with metabolic syndrome and using the same type and dose of GOS, a significant decrease in the total cholesterol/HDL-cholesterol ratio was detected (only after 12 weeks of treatment).^20^ This suggest that the treatment period could be a determining factor in the hypocholesterolemic effect of the GOS. Therefore, the 6 week treatment period of our study could be a limitation, although longer intervention periods could increase the risk of lifestyle changes (diet, physical activity, etc.) as well as favor the withdrawal of the trial. On the contrary, studies carried out in doses of GOS both equivalent to and much higher (5.4–54 g/day, assuming a 65 kg of body weight) than those used in humans during 6–8 weeks reported an improvement in the lipid-related serum parameters (triglycerides, total, HDL-, LDL-, and very low density lipoprotein-cholesterol) in high fat diet-induced metabolic syndrome mice^17^ and dyslipidemia rats^18^ and in healthy rats with a dose–response effect.^16^ Thus, the studies related to the effect of GOS on the serum lipid profile in humans cannot be as conclusive as in murine models.

**Table 1:** Serum Lipid Profile Response upon Regular Consumption of the Beverages (*n* = 42)^a^^b^

	PS-enriched beverage		GOS–PS-enriched beverage	
(mg/dL)	pre-treatment	post-treatment	pre-treatment	post-treatment
total cholesterol	229.8 ± 25.2 a	219.1 ± 22.9 b,x	227.7 ± 25.4 a	216.2 ± 23.8 b,x
LDL cholesterol	142.0 ± 21.1 a	129.2 ± 22.5 b,x	138.9 ± 21.5 a	128.3 ± 18.8 b,x
HDL cholesterol	70.1 ± 14.8 a	71.9 ± 14.8 a,x	71.4 ± 17.7 a	71.0 ± 19.0 a,x

a Results are expressed as mean ± SD.

b Different letters denote significant differences (*p* < 0.05) in the same kind of beverage (PS-enriched or GOS–PS-enriched) between pre-treatment and post-treatment values (a, b) and in the post-treatment values between beverages (x). Reference range (mg/dL): total cholesterol (150–200); LDL cholesterol (70–160); and HDL cholesterol (35–75).

On the other hand, it has been stated that higher baseline LDL-cholesterol concentrations result in greater absolute LDL-cholesterol reductions.^27^ In this regard, we have observed this association among the previous clinical trials carried out with PS-enriched beverages (providing 1.5–2 g PS/day), 129.4 ± 28.5 mg/dL with a 5.1% of reduction^8^ and 146.0 ± 31.8 mg/dL with a 7%,^7^ as well as in the present study (138.9–142 mg/dL with a reduction between 7.6–9%).

#### Serum Sterols

In Table 2, the serum sterol contents and change values (absolute and percentages) are shown. The values have also been normalized with total cholesterol levels in order to compare our results with those previously reported which also applied this method to avoid the interindividual variations in lipoprotein levels. No significant differences in the percentages of the change in PS levels were detected after the consumption of either beverage (without and with GOS addition), suggesting no effect of the presence of GOS on PS bioavailability. These results are in agreement with a previous preliminary *in vitro* study carried out by our research group, in which PS bioaccessibility was not modified by the presence of GOS in milk-based fruit beverages with a similar dosage of enrichment (2.5 g PS and 5.0 g GOS/250 mL).^21^

**Table 2:** Sterol Response in Serum upon Regular Consumption of the Beverages (*n* = 41) (Mean, Confidence Intervals 95%)^a^

	pre-treatment		post-treatment (6 weeks)		change			
sterols	μg mL^–1^	(μmol mmol^–1^ cholesterol)	μg mL^–1^	(μmol mmol^–1^ cholesterol)	absolute (μg mL^–1^)	absolute (μmol mmol^–1^ cholesterol)	(%) (μg mL^–1^)	(%) (μmol mmol^–1^ cholesterol)
PS-Enriched Beverage								
cholestanol	6.68 a (6.27, 7.09)	2.87 a (2.69, 3.05)	6.74 a (6.39, 7.09)	3.03 b (2.87, 3.19)	0.06 y (−0.27, 0.39)	0.16 y (0.01, 0.31)	2.42 y (−2.59, 7.43)	7.18 y (1.75, 12.61)
desmosterol	1.90 a (1.77, 2.03)	0.84 a (0.79, 0.89)	1.96 a (1.81, 2.11)	0.91 b (0.84, 0.98)	0.07 y (−0.02, 0.16)	0.07 y (0.03, 0.11)	4.03 y (−0.34, 8.4)	8.88 y (4.61, 13.15)
lathosterol	3.41 a (2.95, 3.87)	1.49 a (1.29, 1.69)	3.51 a (3.1, 3.92)	1.60 b (1.43, 1.77)	0.10 y (−0.1, 0.3)	0.11 y (0.03, 0.19)	6.10 y (0.13, 12.07)	11.12 y (4.93, 17.31)
total animal sterols	12.28 a (11.53, 13.03)	5.29 a (4.97, 5.61)	12.53 a (11.75, 13.31)	5.64 b (5.31,5.97)	0.25 y (−0.23, 0.73)	0.35 y (0.20, 0.50)	2.61 y (−1.23, 6.45)	7.35 y (3.28, 11.42)
campesterol	4.10 a (3.61, 4.58)	1.72 a (1.53, 1.91)	4.59 b (4.13,5.05)	2.02 b (1.83, 2.21)	0.51 y (0.15, 0.87)	0.30 y (0.15, 0.45)	18.54 y (6.65, 30.43)	23.50 y (11.09, 35.91)
stigmasterol	0.37 a (0.31, 0.43)	0.15 a (0.13, 0.17)	0.40 a (0.34, 0.46)	0.17 b (0.15, 0.19)	0.03 y (−0.01, 0.07)	0.02 y (0.01, 0.04)	15.53 y (1.33, 29.73)	21.03 y (6.48, 35.58)
β-sitosterol	4.39 a (3.96, 4.82)	1.79 a (1.62, 1.96)	5.63 b (5.12, 6.14)	2.40 b (2.20, 2.60)	1.24 y (0.82, 1.66)	0.61 y (0.44,0.78)	32.48 y (22.01, 42.95)	38.75 y (27.77,49.73)
total PS	8.79 a (7.92, 9.66)	3.62 a (3.28, 3.96)	10.46 b (9.54, 11.38)	4.51 b (4.15, 4.87)	1.67 y (0.97, 2.37)	0.89 y (0.61, 1.17)	22.81 y (13.29, 32.33)	28.73 y (18.69, 38.77)
GOS–PS-Enriched Beverage								
cholestanol	6.67 a (6.31, 7.03)	2.90 a (2.73, 3.07)	6.73 a (6.33, 7.13)	3.06 b (2.88, 3.24)	0.06 y (−0.16, 0.28)	0.16 y (0.05, 0.27)	1.05 y (−2.26, 4.36)	6.04 y (2.09, 9.99)
desmosterol	1.92 a (1.76, 2.08)	0.84 a (0.78, 0.90)	1.99 a (1.85, 2.13)	0.92 b (0.86, 0.98)	0.07 y (−0.03, 0.17)	0.08 y (0.03, 0.13)	6.76 y (−0.98, 14.5)	12.50 y (4.35, 20.65)
lathosterol	3.43 a (3.00, 3.86)	1.50 a (1.32, 1.68)	3.50 a (3.07, 3.93)	1.61 b (1.42, 1.80)	0.06 y (−0.12, 0.24)	0.11 y (0.03, 0.19)	4.37 y (−1.74, 10.48)	9.97 y (3.52, 16.42)
total animal sterols	12.30 a (11.62, 12.98)	5.35 a (5.07, 5.63)	12.51 a (11.81, 13.21)	5.70 b (5.40, 6.00)	0.21 y (−0.06, 0.48)	0.35 y (0.20, 0.50)	1.80 y (−0.52, 4.12)	6.79 y (3.91, 9.67)
campesterol	4.23 a (3.74, 4.73)	1.78 a (1.59, 1.97)	4.51 b (4.01, 5.00)	2.00 b (1.79, 2.21)	0.29 y (0.03, 0.55)	0.22 y (0.12, 0.32)	8.03 y (1.79, 14.27)	13.58 y (7.54, 19.62)
stigmasterol	0.38 a (0.32, 0.44)	0.16 a (0.14, 0.18)	0.37 a (0.31, 0.43)	0.16 a (0.14, 0.18)	–0.01 y (−0.05, 0.03)	0.002 y (−0.01, 0.02)	3.98 y (−10.36, 18.32)	10.13 y (−5.67, 25.93)
β-sitosterol	4.65 a (4.12, 5.18)	1.89 a (1.68, 2.10)	5.84 b (5.24, 6.44)	2.51 b (2.26, 2.76)	1.19 y (0.83, 1.55)	0.62 y (0.47, 0.77)	28.88 y (21.39, 36.37)	35.73 y (27.99, 43.47)
total PS	9.19 a (8.21, 10.17)	3.78 a (3.40, 4.16)	10.61 b (9.54, 11.68)	4.67 b (4.23, 5.11)	1.43 y (0.83, 2.03)	0.83 y (0.58, 1.08)	17.33 y (11.01, 23.65)	23.57 y (17.10, 30.04)

a Analyses were made in triplicate. Different letters denote significant differences (*p* < 0.05) in the same kind of beverage (PS-enriched or GOS–PS-enriched beverage) among pre-treatment and post-treatment values (within lines) (a,b) or in different beverages among changes (absolute or expressed as percentage) (within columns) (y,z). Absolute change = post-treatment level minus pre-treatment level. Change (%) = absolute change × 100/pre-treatment level. Total PS: sum of campesterol, stigmasterol, and β-sitosterol.

The regular intake of the beverages significantly increased normalized concentrations of campesterol (13.6–23.5%) and β-sitosterol (35.7–38.8%) as markers of dietary PS intake. Stigmasterol (minor PS in the beverage) only increased in the PS-enriched beverage. In this study, serum levels of PS cannot be considered as markers of cholesterol absorption since the intake of dietary PS increased due to intervention.^28^

Similarly to the PS contents, no differences in cholesterol metabolism markers were observed between treatments. Cholestanol, desmosterol, and lathosterol showed increases of 6.0–7.2, 8.9–12.5, and 10.0–11.1%, respectively, after intervention. On comparing our results with a previous clinical study,^8^ we find that similar significant increases of desmosterol, lathosterol, campesterol, and β-sitosterol were obtained with a close PS-enriched beverage. Although in the present study, there was a significant change in stigmasterol contents with respect to the aforementioned clinical trial; the absolute increments (in μg/mL, 0.03 *vs* 0.00) cannot be considered relevant as it presented a low concentration in the beverages and a low absorption.

The decrease (*p* < 0.05) in total cholesterol levels obtained during the intervention was not reflected in a drop of cholestanol (cholesterol absorption marker), but there was a significant increase in desmosterol and lathosterol (cholesterol synthesis markers) (Table 2). In agreement with our results, an increase in serum cholesterol synthesis markers in postmenopausal women with mild hypercholesterolemia who intake PS-enriched margarine has been observed,^29^ although they also reported a decrease in cholestanol levels. It could be assumed that with higher intervention times (6 months *vs* 6 weeks), the different types and doses of PS (3 g of plant stanol ester *vs* 2 g of free phytosterols) could partly justify these differences.

### Impact of GOS on Colonic Sterol Metabolization

#### Fecal Animal Sterols

Fecal animal contents for the two sampling points (pre-treatment and posttreatment) as well as the absolute change from basal values after regular consumption of the PS or GOS–PS-enriched beverages are shown in Table 3.

**Table 3:** Fecal Animal Sterol Contents (mg/g Freeze-Dried Feces) after Regular Consumption of the Beverages (*n* = 42) (Median, Percentile 25–75%)^a^

						conversion percentages	
sterol	pre-treatment	post-treatment (6 weeks)	*p* value	absolute change	*p* value	low converters	high converters
PS-Enriched Beverage							
cholesterol	2.19 (1.48; 2.76) a	3.94 (1.99; 5.58) b	2 × 10^–4^	1.43 (0.04; 3.09) y		30.3–36.0 (2)	51.3–93.8 (40)
coprostanol	13.38 (9.62; 18.71) a	10.68 (6.74; 15.89) b	8 × 10^–4^	–3.16 (−5.35; −0.47) y			
coprostanone	0.93 (0.40; 2.28) a	1.67 (0.96; 3.11) b	2 × 10^–3^	0.54 (−0.27; 1.22) y			
cholestanol + methylcoprostanol^b^	1.15 (0.93; 1.42) a	1.77 (1.23; 2.76) b	3 × 10^–6^	0.62 (0.16; 1.41) y			
lathosterol	0.09 (0.07; 0.12) a	0.09 (0.07; 0.13) a	0.40	0.01 (−0.01; 0.02) y			
total animal sterols	19.28 (13.49; 26.24) a	20.05 (13.29; 27.10) a	0.65	–0.01 (−3.33; 5.40) y			
GOS–PS-Enriched Beverage							
cholesterol	1.90 (1.49; 3.03) a	3.99 (2.33; 6.05) b	7 × 10^–6^	1.35 (0.43; 3.79) y	0.38	2.1–44.1 (3)	51.0–94.3 (39)
coprostanol	14.45 (10.96; 18.64) a	12.07 (7.06; 15.26) b	4 × 10^–3^	–2.05 (−7.51; 0.70) y	0.96		
coprostanone	0.87 (0.47; 2.14) a	2.34 (1.15; 3.13) b	7 × 10^–4^	0.76 (−0.17; 2.24) y	0.45		
cholestanol + methylcoprostanol^b^	1.02 (0.90; 1.35) a	1.84 (1.33; 2.37) b	7 × 10^–7^	0.78 (0.21; 1.29) y	0.48		
lathosterol	0.09 (0.07; 0.12) a	0.09 (0.07; 0.15) a	0.11	0.01 (−0.02; 0.03) y	0.50		
total animal sterols	19.58 (16.30; 25.90) a	22.49 (17.66; 27.91) a	0.65	1.59 (−3.21; 5.84) y	0.67		

a Absolute change: post-treatment level minus pre-treatment level. Different letters denote significant differences (*p* < 0.05) in the same kind of beverage (PS-enriched or GOS–PS-enriched) among pre-treatment and post-treatment values (within lines) (a,b), or in different beverages among absolute changes (within columns) (y,z). Cholesterol conversion percentage: [coprostanol + coprostanone/(cholesterol + coprostanol + coprostanone)] × 100. Low and high converters were defined according to Wilkins & Hackman (1974)^36^ considering that low converters have a sterol conversion rate of <50% and high converters of >50%. The number of subjects corresponding to each group is indicated in parentheses.

b The applied method does not allow the separation of these compounds.

The total fecal animal sterol contents ranged from 13.29 to 27.10 mg/g freeze-dry feces, which are in line with those obtained in a previous study of our research group (13.9–30.10 mg/g freeze-dry feces).^14^ Coprostanol was the major metabolite, accounting for 53–54% of the total animal sterols after treatment. No statistically significant differences (*p* < 0.05) were observed after the intake of the beverages (neither with respect to the baseline/pre-treatment value or between absolute change for both beverages).

After intake of PS- or GOS–PS-enriched beverages, a significant increase (post-treatment *vs* pre-treatment) in the excretion of cholesterol (65 and 71%), coprostanone (58 and 87%), and cholestanol + methylcoprostanol (54 and 76%) was observed. A significant decrease in coprostanol (24 and 14%) and no change in the lathosterol content were also reported. It should be noted that although in the presence of GOS, these changes are more pronounced and there are no differences between the absolute changes of individual animal sterols for both beverages (see Table 3).

The increase of cholesterol fecal excretion is a fact already indicated in a previous study by our group after the intake of a beverage enriched in PS (2 g/day) similar to that administered in this study.^14^ It can be justified by the known interaction of PS in the absorption of cholesterol. In addition, this work confirms again that in the presence of large amounts of PS, the metabolism of cholesterol by the microbiota occurs through an incomplete indirect route which results in greater excretion of coprostanone and less coprostanol or another pathway, in which cholestanol is formed by reduction of cholestenone and cholestanone.^30^ The intake of margarine containing PS (8.6 g/day) by normolipidic subjects during 28 days also reduces the metabolism of cholesterol to coprostanol.^12^

#### Fecal PS

In contrast to total animal sterols, significant increases (*p* < 0.05) are observed in total PS after the intake of both beverages with respect to pre-treatment values (23.31–55.94 *vs* 4.96–7.58) (see Table 4). These results reflect women’s adherence to the study, although no influence of GOS on the absolute changes is observed (see Table 4). These facts also occur in individual sterols, mainly increases in ethylcoprostanol (post-treatment: 45–48% of the total PS and derived from β-sitosterol) and methylcoprostanone (from campesterol).

**Table 4:** Fecal PS Contents (mg/g Freeze-Dried Feces) after Regular Consumption of the Beverages (*n* = 42) (Median, Percentile 25–75%)^a^

						conversion percentages	
sterol	pre-treatment	post-treatment (6 weeks)	*p* value	absolute change	*p* value	low converters	high converters
PS-Enriched Beverage							
β-sitosterol	0.74 (0.64; 0.99) a	11.21 (2.29; 22.33) b	2 × 10^–7^	8.29 (1.49; 17.27) y		9.4–49.5 (15)	50.0–87.8 (26)
sitostanol	0.57 (0.46; 0.66) a	3.33 (1.99; 5.74) b	5 × 10^–8^	2.84 (1.34; 4.91) y			
ethylcoprostanol	3.97 (2.70; 5.17) a	17.49 (7.36; 28.66) b	8 × 10^–8^	12.95 (2.65; 20.66) y			
campesterol	0.32 (0.24; 0.43) a	1.57 (0.62; 2.40) b	2 × 10^–7^	1.09 (0.34; 2.03) y		0.5–44.3 (42)	
campestanol	0.38 (0.31; 0.50) a	0.86 (0.58; 1.31) b	4 × 10^–8^	0.46 (0.20; 0.80) y			
methylcoprostanone	0.05 (0.03; 0.11) a	0.24 (0.07; 0.43) b	2 × 10^–6^	0.15 (0.03; 0.40) y			
stigmasterol	0.06 (0.04; 0.08) a	0.16 (0.08; 0.26) b	1 × 10^–5^	0.07 (0.00; 0.19) y		0.0002–48.6 (30)	50.9–90.3 (12)
ethylcoprostenol	0.11 (0.08; 0.13) a	0.10 (0.08; 0.13) a	0.93	0.002 (−0.02; 0.02) y			
total PS	6.77 (4.96; 7.58) a	36.49 (23.27; 53.89) b	2 × 10^–10^	29.05 (11.77; 44.55) y			
GOS–PS-Enriched Beverage							
β-sitosterol	0.76 (0.56; 1.13) a	12.02 (2.68; 20.36) b	3 × 10^–8^	10.79 (2.14; 19.30) y	0.79	1.5–45.1 (15)	51.2–85.8 (26)
sitostanol	0.56 (0.50; 0.73) a	3.90 (1.98; 5.42) b	3 × 10^–8^	3.16 (1.27; 4.80) y	1.00		
ethylcoprostanol	3.47 (2.47; 4.58) a	17.40 (8.65; 27.32) b	5 × 10^–8^	14.47 (4.91; 21.56) y	0.88		
campesterol	0.31 (0.23; 0.42) a	1.60 (0.87; 2.55) b	1 × 10–10	1.41 (0.47; 2.11) y	0.64	0.7–49.0 (42)	
campestanol	0.36 (0.26; 0.46) a	0.83 (0.59; 1.23) b	8 × 10^–8^	0.44 (0.23; 0.82) y	0.61		
methylcoprostanone	0.05 (0.03; 0.08) a	0.25 (0.12; 0.50) b	1 × 10^–6^	0.18 (0.03; 0.45) y	0.36		
stigmasterol	0.06 (0.05; 0.09) a	0.18 (0.09; 0.30) b	8 × 10^–8^	0.09 (0.02; 0.23) y	0.67	0.0002–47.9 (29)	51.9–83.7 (13)
ethylcoprostenol	0.10 (0.09; 0.12) a	0.11 (0.09; 0.13) a	0.37	0.01 (−0.01; 0.02) y	0.25		
total PS	6.01 (5.01; 7.26) a	38.99 (23.21; 55.94) b	4 × 10^–8^	32.54 (18.07; 49.28) y	0.60		

a Absolute change: post-treatment level minus pre-treatment level. Different superscript letters denote significant differences (*p* < 0.05) in the same kind of beverage (PS-enriched or GOS–PS-enriched) among pre-treatment and post-treatment values (within lines) (a,b) or in different beverages among absolute change (within columns) (y,z). β-Sitosterol conversion percentage: [ethylcoprostanol/(β-sitosterol + sitostanol + ethylcoprostanol)] × 100; campesterol conversion percentage: [methylcoprostanone/(campesterol + campestanol + methylcoprostanone)] × 100; stigmasterol conversion percentage: [ethylcoprostenol/(stigmasterol + ethylcoprostenol)] × 100. Low and high converters were defined according to Wilkins & Hackman (1974)^36^ considering that low converters have a sterol conversion rate of <50% and high converters of >50%. The number of subjects corresponding to each group is indicated in parentheses.

#### GOS and Sterol Metabolism Interaction

**Figure 4:**
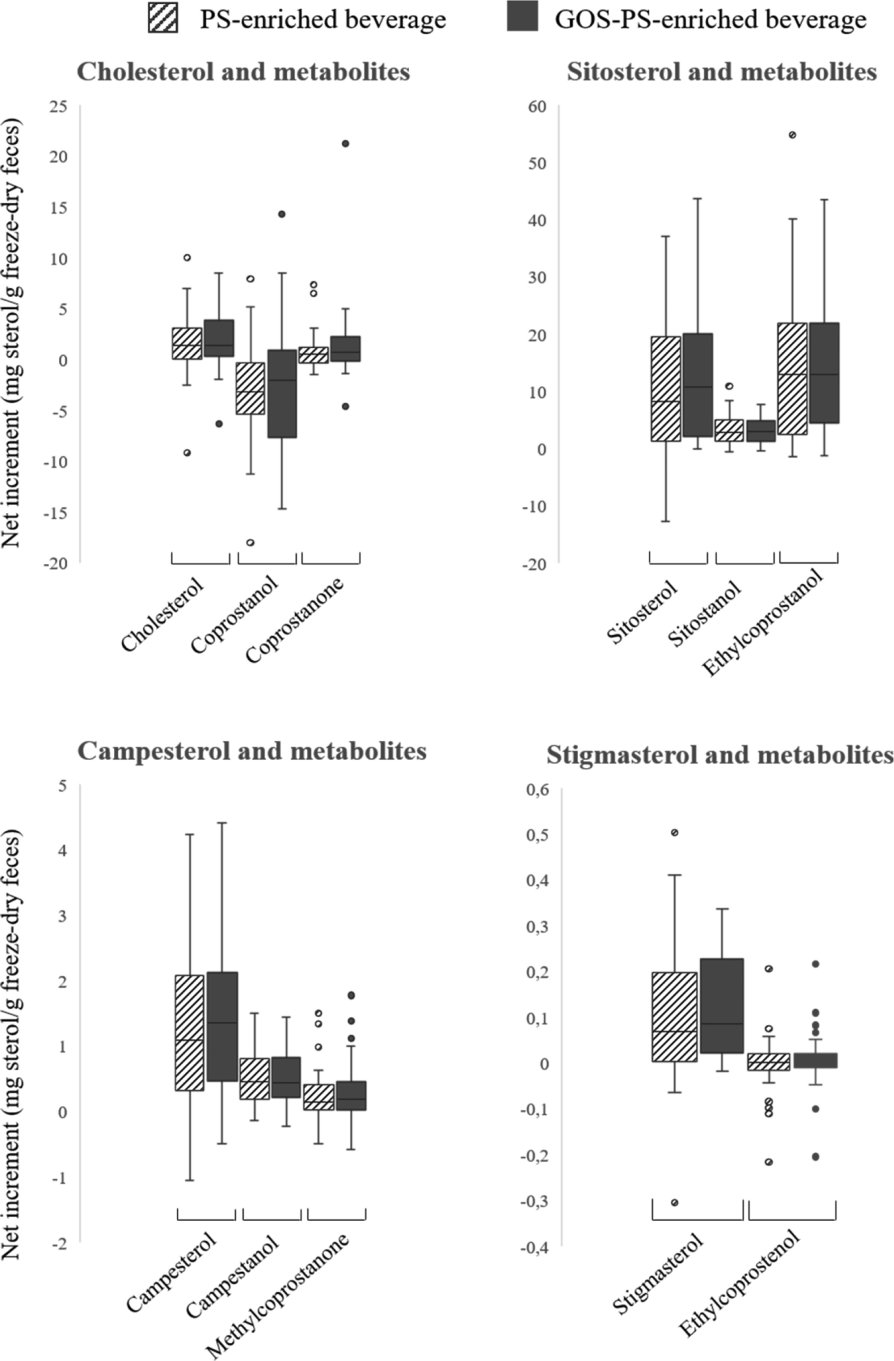
Sterol response in feces upon regular consumption of beverages (*n* = 42). Boxes represent the mean of the absolute change (post-treatment – pre-treatment values). Points in each box represent outlier values.

No influence of GOS on colonic fermentation of cholesterol and PS has been observed, and this fact is well displayed on the boxes, which represent the mean of the absolute change after consumption of PS- or GOS–PS-enriched beverages (see Figure 4). In both cases, the response of the women was very similar, observing very few outliers despite the fact that cholesterol and PS intake from the diet was not controlled (which constitutes a limitation of the study). The absence of effect of GOS enrichment upon sterol metabolism disagrees with what was observed in a novel *in vitro* approach carried out by our research group. During dynamic colonic fermentation of a PS-enriched beverage (2.5 g/250 mL) for one week, an absence of sterol metabolites was observed.^31^ In contrast, in the same kind of beverage also enriched with GOS (4.5 g/250 mL), a rapid sterol metabolization (indicated by the absence of intermediary metabolites) was determined in the vessels corresponding to the transverse and descending sections of the colon.^32^ Moreover, beverage fermentation in the presence of GOS showed higher ratios of cholesterol biotransformation compared to PS and that the direct pathway of conversion to coprostanol is predominant (in contrast with the abovementioned results), suggesting that GOS promotes its metabolization. However, these *in vitro* studies also reveal a close relation between microbiota composition and sterol metabolism. In the case of the *in vitro* dynamic fermentation of GOS–PS-enriched beverage,^32^ sterol metabolites were only observed in those compartments of the colon with a more similar microbial community (transverse and descending colon reactors). For that reason, other limitation of our study was the lack of microbiota analysis, which would have provided valuable information about the absence of effect of GOS and other bioactive compounds (polyphenols) present in the beverage on sterol metabolism. As indicated above, the hypothesis of the present study suggests that the addition of GOS to the beverage could alter sterol metabolism through modification of the microbiota composition (see Figure 1). In this sense, GOS could modulate the *Bifidobacterium* population (as they are highly specific in promoting its growth) as well as other bacterial species, also considering coprostanoligenic (*Bacteroides* or *Eubacterium* species). Note that *Eubacterium* is the only species associated with the PS biotransformation pathway.^11^ The results obtained in the present study suggest that this bifidogenic effect and/or modification of microbial species related to sterol metabolism did not occur due to the absence of differences between treatments (GOS–PS- *vs* PS-enriched beverages). This is partially coincident with the results obtained during the *in vitro* dynamic colonic fermentation study, in which no bifidogenic effect nor other modulation in species population related to sterol metabolism was determined. However, the production of metabolites during the fermentation assay suggests the involvement of other so far unidentified bacterial species in the sterol metabolization pathway. In this sense, although the bifidogenic effect of GOS is widely reported in *in vivo* studies,^15^ certain investigations have shown a minimum dose of 5 g GOS/day (slightly higher than the one used in the present study) to achieve significant increases in bifidobacteria counts.^33−35^ Differences in GOS ingredient, delivery vehicle, experimental design, and microbiota methods of analysis used could also influence the variations in the expected bifidogenic effect.^34^

#### Sterol Conversion Percentages and Metabolic Capacity

In order to know if the intake of the beverages enriched with GOS modifies the biotransformation of cholesterol and individual PS (β-sitosterol, stigmasterol, and campesterol), conversion percentages have been calculated and women have been classified as low or high converters according to Wilkins & Hackman (1974)^36^ (see Tables 3 and 4). Independent of the beverages ingested, women are predominant high converters of cholesterol (*n* = 39 or 40) and β-sitosterol (*n* = 26). However, all subjects are low converters of campesterol and, in general, also of stigmasterol. These results are only partially in line with a previous study,^14^ which presented a lower number of high cholesterol converters (29) and β-sitosterol (17) and a higher number of high stigmasterol converters (27 *vs* 13). Note that it is known that the efficiency of microbial cholesterol-to-coprostanol conversion in human populations (members of genus *Eubacterium* and strains of *Bifidobacterium*, *Lactobacillus,* and *Peptostreptococcus*) is a majority of high converters.^37^ The intake of the GOS-enriched beverage induces slight changes in the women’s colonic metabolic capacity (see Supporting Information Figure S1). The conversion capacity of cholesterol and stigmasterol in 12 women is reduced by 10% and of β-sitosterol by 16%. Only in five of them, the metabolism of all these sterols reduced simultaneously. A decrease in cholesterol conversion percentage after the intake of a similar PS-enriched beverage has been previously reported,^14^ associated with the increase in PS consumption that could reduce or block cholesterol metabolite production. Reduction of cholesterol conversion is the interest since cholesterol metabolites are associated with procarcinogenic action and could increase the risk of colon cancer.^11^ The different responses to the presence of GOS in colonic sterol metabolism could also be derived from the different phylogenetic properties of each woman’s microbiota. It has been reported that composition of the microbiota can be stratified into three main groups (enterotypes), which have functional differences with respect to how they obtain energy from the substrates available in the colon. In this sense, subjects belonging to enterotype 1 or *Bacteroides* have been shown to obtain energy primarily from the fermentation of carbohydrates and proteins, whereas enterotype 2 and 3 (*Prevotella* and *Ruminococcus*, respectively) are more efficient in degrading mucin.^38^

As coprostanol has low intestinal absorption, it has been proposed that a high conversion efficiency of cholesterol to coprostanol could improve the serum lipid profile by facilitating the removal of cholesterol from the body.^39^ In this sense, Sekimoto et al.^40^ observed an inverse correlation between serum cholesterol levels and fecal coprostanol/cholesterol ratio, suggesting that coprostanol production could modulate cholesterol blood levels. Recently, a clinical trial involving overweight postmenopausal women also showed an inverse correlation between the fecal coprostanol/cholesterol ratio and serum total and LDL-cholesterol after an intervention of 4 weeks using milk polar lipids (3–5 g/day).^41^ However, in the present study, this correlation has not been observed for any beverage nor between serum cholesterol levels and cholesterol conversion percentages in feces. The absence of this correlation could be due to the presence of PS at an enrichment dose. As mentioned above, in this case, the direct metabolization pathway of cholesterol to coprostanol is modified promoting the formation of intermediary metabolites in detriment of coprostanol levels.

Therefore, although the results of the present clinical trial carried out with a regular intake of two PS-enriched beverages with or without GOS were not as expected in the first approach, valuable information has been obtained:

(i) GOS do not modify the bioavailability of PS and do not enhance its blood cholesterol-lowering effect.
(ii) It is confirmed that ingestion of PS from enriched food modulates fecal excretion of sterols and metabolites.
(iii) Sterol colonic metabolism was not altered by the addition of GOS to the beverage, a fact that is reflected with slight changes in women’s colonic metabolic capacity. This is probably due to the lack of effect of the prebiotics on coprostanoligenic bacteria, and presumably, longer intervention times (over 12 weeks) are required to verify any beneficial effect.

Even though many trials have been carried out in this regard, the results are at times contradictory and, therefore, prebiotic supplementation and its relationship with blood lipid levels warrant further research. In future studies, factors that limit this work should be controlled, such as individual’s genotype and lifestyle (physical exercise), cholesterol and PS content of the diet, and the changes exerted upon microbiota composition.

## Supporting Information Available

Graphic of sterol conversion percentages (PDF)

## Abbreviations

β-Cx: β-cryptoxanthin
BMI: body mass index
FFQ: food frequency questionnaire
GOS: galactooligosaccharides
MFGM: milk fat globule membrane
PS: plant sterols
