# Supplementary material for: Influence of Galactooligosaccharides on the Positive Effect of Plant Sterol-Enriched Beverages on Cardiovascular Risk and Sterol Colon Metabolism
Source: J Agric Food Chem. 2022 Jan 11;70(2):532–42. doi: 10.1021/acs.jafc.1c06120 (PMC9127961; doi:10.1021/acs.jafc.1c06120)
Supplement: Supplementary file 1 — jf1c06120_si_001.pdf [file jf1c06120_si_001.pdf]

**Figure S1.** Sterol conversion percentages after beverages consumption (*n = 42*)

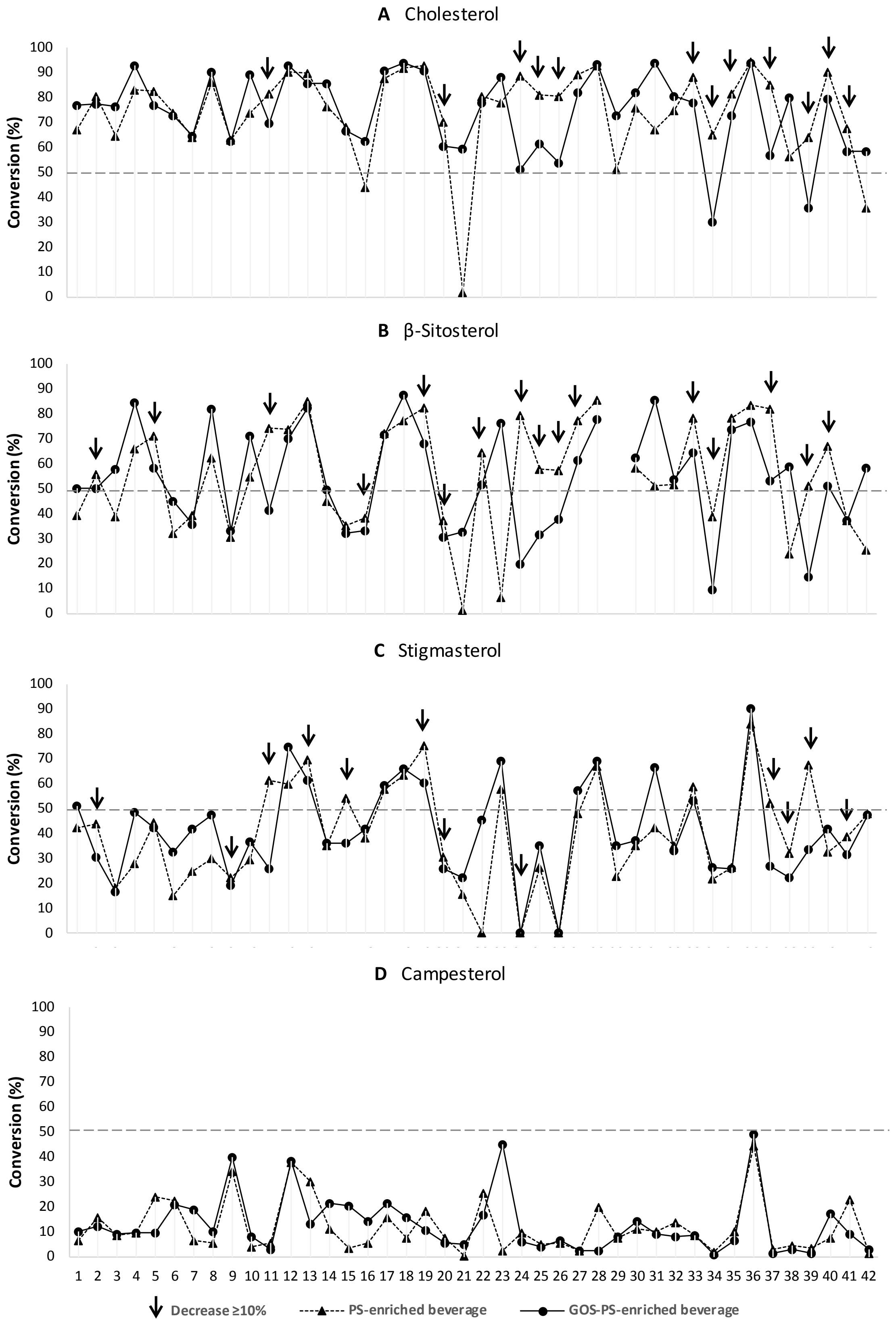


Abscissa axis represents the 42 postmenopausal women from the clinical study, whereas ordinate axis represents the sterol conversion percentage. Cholesterol conversion percentage: [coprostanol + coprostanone/(cholesterol + coprostanol + coprostanone)] x 100. β-Sitosterol conversion percentage: [ethylcoprostanol/(β-sitosterol + sitostanol +

ethylcoprostanol)] x 100; Stigmasterol conversion percentage: [ethylcoprostenol/(stigmasterol + ethylcoprostenol)] x 100; Campesterol conversion percentage: [methylcoprostanone/(campesterol + campestanol + methylcoprostanone)] x 100. To classify low and high converters, a dashed line was located on each graph indicating the threshold of 50% (according to Wilkins & Hackman, 1974). Black arrows indicate a significant decrease of sterol conversion percentage (≥10%) after PS-GOS-enriched beverage intake compared to PS-enriched beverage.
